# Comparative efficacy and safety of licensed treatments for previously treated non-small cell lung cancer: A systematic review and network meta-analysis

**DOI:** 10.1371/journal.pone.0199575

**Published:** 2018-07-25

**Authors:** Xavier Armoiry, Alexander Tsertsvadze, Martin Connock, Pamela Royle, G. J. Melendez-Torres, Pierre-Jean Souquet, Aileen Clarke

**Affiliations:** 1 University of Warwick, Warwick Medical School, Division of Health Sciences, Coventry, England; 2 School of Epidemiology and Public Health, University of Ottawa, Ottawa Canada; 3 Hospices Civils de Lyon, Groupement hospitalier Sud, Service de Pneumologie oncologique, Pierre-Bénite, France; Universidade do Algarve Departamento de Ciencias Biomedicas e Medicina, PORTUGAL

## Abstract

**Purpose:**

This systematic review with network meta-analysis compared the efficacy and safety of currently licensed second-line treatments in patients with late stage non-small cell lung cancer (NSCLC).

**Methods:**

Randomised controlled trials (RCTs) of participants with advanced/metastatic NSCLC receiving second/third line treatments were screened. We searched electronic databases (MEDLINE; EMBASE; Web of Science) from January, 2000 to July, 2017.

Two reviewers screened bibliographic records, extracted data, and assessed risk of bias of included studies. The outcomes were overall survival (OS), progression-free survival (PFS), and drug-related grade 3–5 adverse-events (AEs). We pooled study-specific hazard ratios (HR; for OS and PFS) and risk ratios (RR; for AEs) using conventional and network-meta-analyses, and ranked interventions by the surface under the cumulative ranking curve.

**Findings:**

We included 11 RCTs (7,581 participants) comparing nine drugs. All drugs except for erlotinib significantly improved OS compared to docetaxel. Nivolumab was the highest ranking drug followed by atezolizumab and pembrolizumab. There was no significant difference in OS across these three drugs (HR = 0.98, 95% CI 0.79, 1.21 for nivolumab vs atezolizumab; HR = 0.98, 95% CI 0.77, 1.25 for nivolumab vs pembrolizumab). For PFS, ramucirumab + docetaxel and nivolumab were the drugs with the highest ranking. All interventions except ramucirumab + docetaxel had a reduced risk for severe drug-related AEs vs. docetaxel. Of the drugs with the highest ranking on AEs, nivolumab was significantly safer compared to atezolizumab (RR = 0.55, 95% CI 0.38, 0.79) or pembrolizumab (RR = 0.52, 95% CI 0.34, 0.81).

**Implications:**

Nivolumab, pembrolizumab and atezolizumab exhibited superior benefit/risk balance compared to other licensed drugs used late stage NSCLC. Our results indicate that the use of immunotherapies in people diagnosed with non-specific late stage NSCLC should be promoted. The use of docetaxel may now be judged irrelevant as a comparator intervention for approval of new drugs for second line treatment of NSCLC.

**Study registration number:**

PROSPERO CRD42017065928.

## Introduction

Lung cancer remains one of the most common cancers worldwide [1], with non-small cell lung cancer (NSCLC) accounting for 85 to 90% of all forms of lung cancer.[2] Because NSCLC is predominantly diagnosed at a late stage, most patients are not eligible for otherwise curative surgery, and thus have poor prognoses. While many first-line chemotherapies are available for patients with advanced/ metastatic NSCLC, second-line therapeutic options have been limited to docetaxel.[3] The development of targeted therapies and immunotherapies promises to fill some of the unmet need for the treatment of advanced/ metastatic NSCLC. In 2017, 13 agents had a label indication for the treatment of advanced/ metastatic NSCLC in patients after failure to respond to first-line chemotherapy. This includes three immune checkpoints (nivolumab, pembrolizumab, and atezolizumab). Although the effectiveness and safety of these drugs have been compared to those of docetaxel, they have not been compared to each other head-to-head.

In this systematic review and network meta-analysis (NMA), we compared the clinical efficacy and safety of the agents according to their licensed indication in patients with NSCLC (free of anaplastic lymphoma kinase [ALK] positive and Epidermal growth factor receptor [EGFR] positive expression) for whom first-line treatments failed.

## Methods

We registered a protocol for this review in PROSPERO (CRD42017065928) (Study protocol in S1 File; Prisma checklist in S2 File).

### Eligibility criteria: Studies, participants, and interventions

We included randomised controlled trials (RCTs) of people with advanced or metastatic (IIIB or IV) NSCLC of squamous, non-squamous, or mixed histology who experienced failure to prior first-line chemotherapy. Study populations had to have negative or predominantly negative expressions of ALK and EGFR. Patients with ALK and/or EGFR positive expression were ineligible, since they would be offered targeted therapies (e.g., erlotinib, gefitinib, osimertinib, crizotinib, or ceretinib).[1]

The interventions of interest were the drugs with a European Medicines Agency (EMA) () label indication for the population described above as of June, 2017: Docetaxel (DOC), Pemetrexed (PEM), Ramucirumab plus docetaxel (RAM + DOC), Erlotinib (ERL), Nintedanib plus docetaxel (NINTE + DOC), Afatinib (AFA), Nivolumab (NIVO), Pembrolizumab (PEMBRO), and Atezolizumab (ATEZO). The efficacy outcomes assessed were overall survival (OS), progression-free survival (PFS), the proportion of patients reporting at least one drug-related grade 3 to 5 adverse event (AE), and the proportion of patients discontinuing study medication due to a drug-related AE.

### Search strategy and study selection

English language studies were searched in databases (MEDLINE; EMBASE; Web of Science) from January, 2000 to July, 2017 (Supplementary online material A in S3 File).

Reference lists of relevant studies were scanned to identify additional citations. We consulted the EMA website to identify trials submitted by manufacturers in support of included drugs and sought relevant conference abstracts via relevant web sites.

Three reviewers (X.A., A.T., & M.C.) independently screened all titles/abstracts and examined full-text publications of potentially relevant citations. Disagreements were discussed and resolved through consensus. The study flow and reasons for exclusion at the full-text level were documented in the Preferred Reporting Items for Systematic Reviews and Meta-Analyses (PRISMA) flow-chart. [4]

### Review outcomes and data extraction

Two reviewers (X.A. & A.T.) independently extracted relevant data using an *a priori* defined pre-piloted extraction sheet. Data extracted included study author, country, funding source, sample size, patient characteristics (age, sex, diagnosis, data on tumour stage/histology), type, mode, dose and duration of treatments, dropouts, efficacy/safety outcomes of interest. The data extracted were cross-checked and any disagreements were resolved by discussion or recourse to another reviewer (M.C.).

For each study, we ascertained the estimates of hazard ratio (HR) for OS and PFS and risk ratios (RR) for drug-related grade 3 to 5 AEs, and discontinuation of study medication due to drug-related AE with corresponding 95% confidence intervals (95% CI). We extracted the HRs as reported in the primary studies. These were all derived from Cox regression stratified according to strata specified for randomisation. HRs adjusted for variables additional to randomisation strata were not included in the NMA. If time to progression (TTP) was reported, but not PFS, we used the TTP HR as a proxy for PFS HR. We used “treatment-emergent AEs” as a proxy for drug-related grade 3 to 5 events, if the latter was not reported.

When study results were available for different follow-ups, we extracted the outcomes from the latest follow-up irrespective of the publication type. To address incomplete reporting of outcomes, we used methods published by Tierney et al. [5] and by Guyot et al. [6]

### Risk of bias assessment

Two reviewers independently assessed the risk of bias (RoB) (per outcome: OS and PFS) using the Cochrane RoB tool (Details in Supplementary online material B in S3 File). [7]

### Data synthesis and analysis

Study and population characteristics were summarised in text and evidence tables. Where possible, analyses were stratified by histologic subtypes (squamous and non-squamous) and tumour stage. The analyses included patients with adenocarcinoma but not those with non-squamous histology [8, 9], or where the licensed indication was only for adenocarcinoma [10] in the non-squamous analyses. The label indication for PEM specifies NSCLC “other than predominantly squamous histology,” hence PEM was excluded from squamous analyses. For PEMBRO [9], we analysed data from the licenced 2mg/kg arm.

We used pairwise random-effects meta-analysis to pool the study-specific estimates with 95% CIs. The heterogeneity across trials was examined by visual inspection of forest plots and I^2^ statistics (I^2^>50% indicating a substantial degree of heterogeneity). Sensitivity analyses were planned to assess the robustness of effect estimates across two RoB domains: allocation concealment and blinding of outcome assessors.

We assessed the transitivity assumption [11] by examining the distribution of the effect modifiers across studies (age, sex, performance status, stage IIIB vs IV at inclusion, and number of prior lines) and the dosages of common comparators used as anchor(s). Where possible, we planned to use a node-splitting test within each network with a loop to assess inconsistency between direct and indirect evidence. [12]

We undertook random-effects network meta-analyses in the frequentist framework. Where there were few studies for each contrast between two treatments, we used a fixed- effect model. Summary league tables were generated for all comparisons. [13]

We generated the surface under the cumulative ranking curve (SUCRA) to rank each intervention (i.e., probability of an intervention being superior in effectiveness or safety compared to DOC). [13]

Clustered ranking plots for efficacy/safety outcomes were produced. [14] The threshold for the statistical significance was chosen as a two-tailed alpha = 0.05. All statistical analyses were performed using Stata^®^ version 14.2 (StataCorp, USA).

## Results

**Fig 1:**
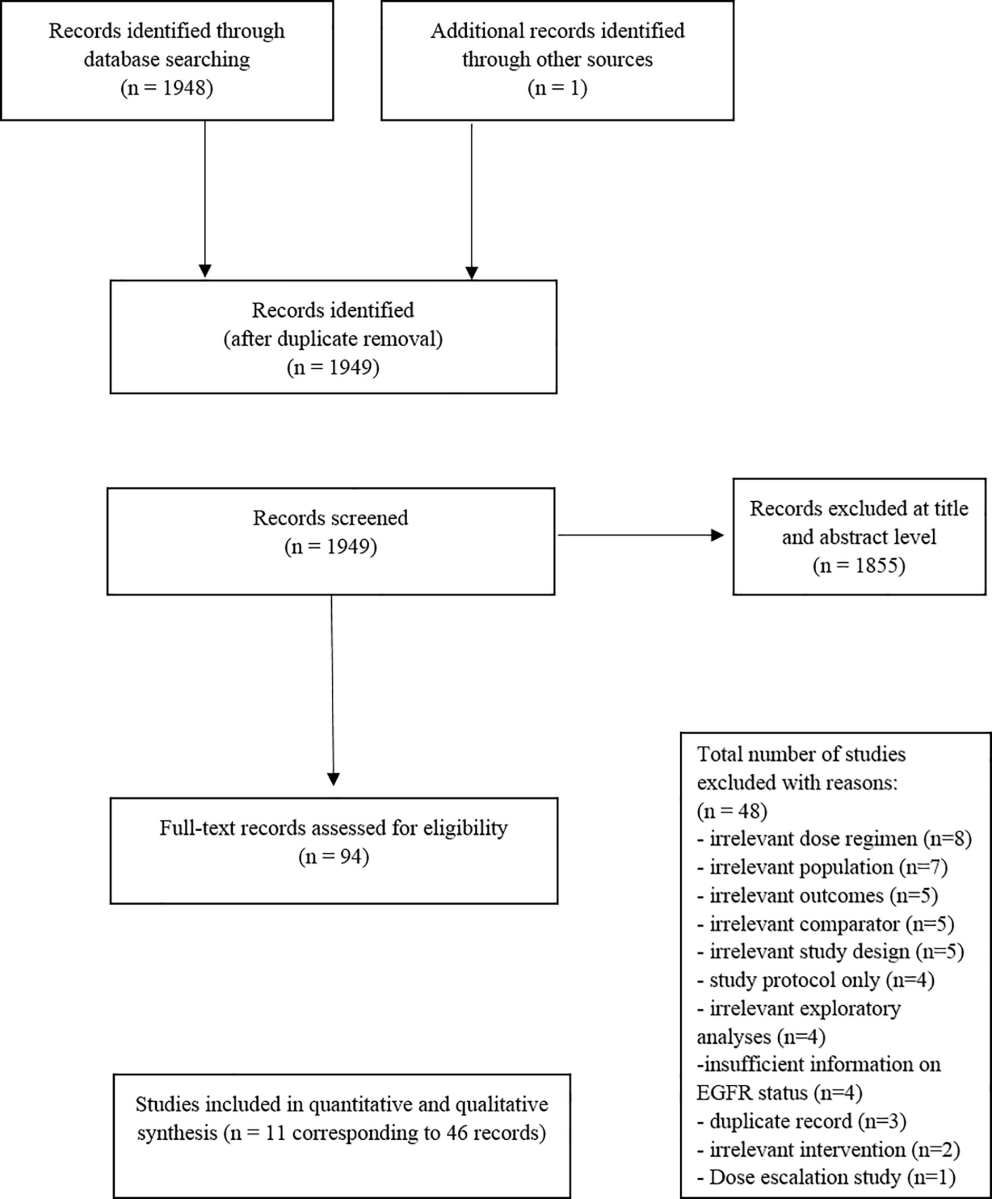
PRISMA flowchart for study selection.

Of 1,949 records identified and screened at title/abstract level, 94 were examined for full-text, of which 46 records [8–10, 15–57] corresponding to 11 RCTs with a total of 7,581 participants were included (Fig 1).

Among the 46 records, 31 [15–45] were supplementary sources of the main publications and three [46, 52, 56] were conference abstracts presenting updated results from primary publications. [8–10, 47–51, 53–55, 57]

### Characteristics of included studies

The mean age at inclusion in the eleven RCTs ranged from 57 to 66 years with a majority of male participants. The sample size ranged from 219 [8] to 1314 [10] patients. All studies included predominantly people with stage IV NSCLC and performance status 1. Only two studies had histology-specific inclusion criteria. [47, 48]

The included RCTs compared nine different drugs (AFA, ATEZO, DOC, ERL, NINTE-DOC, NIVO, PEMBRO, PEME, RAMU+DOC), majority of which were compared to DOC. Six RCTs [10, 47, 48, 50, 51, 57] included only people receiving second-line treatment, while four others [9, 49, 53, 54] included those receiving both second- and third-lines. In KEYNOTE-010 [9] (PEMBRO vs DOC) study, patients had tumours expressing PD-L1 with a ≥1% tumour proportion score (TPS) (consistent with the marketing authorisation of PEMBRO). The characteristics of included studies are presented in Table 1.

**Table 1:** Characteristics of included studies.

Variables n (%) unless stated	REVEL		LUME-LUNG 1		CHECKMATE 017		CHECKMATE 057		Hanna		KEYNOTE-010		POPLAR		TAILOR		OAK		Lux-Lung 8		Karampeazis et al. (HORG)	
	RAM + DOC (n = 628)	PBO + DOC (n = 625)	NIN + DOC (n = 655)	PBO + DOC (n = 659)	NIV (n = 135)	DOC (n = 137)	NIV (n = 292)	DOC (n = 290)	PEME (n = 283)	DOC (n = 288)	Pembro (n = 344)	DOC (n = 343)	ATEZ (n = 144)	DOC (n = 143)	ERL (n = 109)	DOC (n = 110)	ATEZ (n = 425)	DOC (n = 425)	AFA (n = 398)	ERL (n = 397)	PEME (n = 166)	ERL (n = 166)
Age, years (median, range)	62 (21–85)	61 (25–86)	60 (53–67)	60 (54–66)	62 (39–85)	64 (42–84)	61 (37–84)	64 (21–85)	59 (22–81)	57 (28–87)	63 (56–69)	62 (56–69)	62 (42–82)	62 (36–84)	66 (40–81)	67 (35–83)	63 (33–82)	64 (34–85)	65 (36–84)	64 (35–88)	66 (42–86)	65 (37–83)
Male sex	419 (67)	415 (66)	476 (73)	479 (73)	111 (82)	97 (71)	151 (52)	168 (58)	194 (68.6)	217 (75.3)	212 (62)	209 (61)	93 (65)	76 (53)	77 (71)	73 (66)	261 (61)	259 (61)	335 (84)	331 (83)	138 (83.1)	135 (81.3)
White	526 (84)	503 (81)	533 (81)	530 (80)	122 (90)	130 (95)	267 (91)	266 (92)	NA	NA	246 (72)	251 (73)	NR	NR	108 (99)	109 (99)	302 (71)	296 (70)	312 (78)	311 (78)	NR	NR
Asian	74 (12)	86 (14)	116 (18)	123 (19%)	4 (3)	2 (1)	9 (3)	8 (3)			73 (21)	72 (21)	NR	NR	1 (1)	1 (1)	85 (20)	95 (22)	86 (22)	86 (22)	NR	NR
Black	17 (3)	16 (3%)	4 (<1)	5 (<1)	6 (4)	2 (1)	7 (2)	9 (3)			13 (4)	7 (2)	NR	NR	0	0	5 (1)	11 (3)	NR	NR	NR	NR
PS 0	207 (33)	199 (32)	187 (29)	189 (29)	27 (20)	37 (27)	84 (29)	95 (33)	251 (88.6)	252 (87.6)	112 (33)	116 (34)	46 (32)	45 (32)	52 (48)	53 (48)	155 (36)	160 (38)	126 (32)	134 (34)	37 (22.3)	44 (26.5)
PS 1	420 (67)	425 (68)	467 (71)	470 (71)	106 (79)	100 (73)	208 (71)	194 (67)			229 (67)	224 (65)	96 (68)	97 (68)	48 (44)	50 (45)	270 (64)	265 (62)	269 (68)	262 (66)	98 (59)	104 (62.7)
Current and former smoker	518 (82)	483 (77)	490 (75)	498 (76)	121 (90)	129 (94)	231 (79)	227 (78)	NA	NA	279 (81)	269 (78)	117 (81)	114 (80)	90 (83)	80 (73)	341 (80)	353 (83)	361 (91)	367 (92)	128 (77.1)	124 (74.7)
Never smoker	109 (17)	141 (23)	165 (25)	161 (24)	10 (7)	7 (5)	58 (20)	60 (21)			63 (18)	67 (20)	27 (19)	29 (20)	19 (17)	30 (27)	84 (20)	72 (17)	26 (7)	18 (5)	24 (14.5)	29 (17.5)
Stage IIIB at inclusion	0	0	148 (23)	146 (22)	29 (21)	24 (18)	20 (7)	24 (8)	71 (25.1)	73 (25.3)	na	na	NR	NR	NR	NR	NR	NR	48 (12)	48 (12)	19 (11.4)	12 (7.2)
Stage IV at inclusion	628 (100)	625 (100)	399 (61)	408 (62)	105 (78)	112 (82)	272 (93)	266 (92)	212 (74.9)	215 (74.7)	na	na	NR	NR	NR	NR	NR	NR	349 (88)	345 (87)	147 (88.6)	154 (92.8)
Non-squamous	465 (74)	447 (72)	347 (53)	352 (53)	0	0	292 (100)	290 (100)	154 (54.4)	142 (49.3)	240 (70)	240 (70)	95 (66)	95 (66)	78 (71.5)	87 (79)	313 (74)	315 (74)	17 (4)	15 (4)	130 (79.3)	127 (76.5)
Squamous	157 (25)	171 (27)	276 (42)	279 (42)	135 (100)	137 (100)	0	0	78 (27.6)	93 (32.3)	76 (22)	66 (19)	49 (34)	48 (34)	31 (28.4)	23 (21)	112 (26)	110 (26)	381 (96)	382 (96)	36 (21.7)	39 (23.5)
Prior platinum-based therapy	623 (99)	622 (99)	628 (97)	636 (98)	135 (100)	138 (100)	292 (100)	290 (100)	262 (92.6)	259 (89.9)	na	na	NR	NR	109 (100)	110 (100)	425 (100)	425 (100)	398 (100)	397 (100)	166 (100)	166 (100)
First-line bevacizumab	88 (14)	92 (15)	27 (4)	23 (4)	1 (1)	2 (1)	na	na	0	0	na	na	NR	NR	NR	NR	NR	NR	NR	NR	NR	NR
Prior maintenance treatment	135 (21)	143 (23%	NA	NA	NA	NA	122 (42)	111 (38)	NA	NA	na	na	144 (100)	143 (100)	109 (100)	109 (99)	NR	NR	NR		NR	NR
Previous taxane	153 (24)	152 (24)	NA	NA	46 (34)	46 (34)	na	na	73 (25.8)	80 (27.8)	na	na	NR	NR	0 (0)	0 (0)	NR	NR	NR	NR	NR	NR
EGFR Wild type	207 (33)	197 (32)	NA	NA	NA	NA	na	na	NA	NA	293 (85)	294 (86)	NR	NR	109 (100)	110 (100)	318 (75)	310 (73)	NR	NR	57 / 62	55 / 61
EGFR Mutant	15 (2)	18 (3)	NA	NA	NA	NA	44 (15)	38 (13)			28 (8)	26 (8)	10 (12)	8 (10)	0	0	42 (10)	43 (10)	NR	NR	5 / 62	6 / 61
Unknown or missing	406 (65)	410 (66)	NA	NA	NA	NA	na	na			23 (7)	23 (7)	NR	NR	0	0	65 (15)	72 (17)	NR	NR	NR	NR
1 prior therapy	628 (100)	625 (100)	655 (100)	659 (100)	135 (100)	137 (100)	292 (100)	290 (100)	283 (100)	288 (100)	243 (71)	235 (69)	93 (65)	96 (67)	Unclear	unclear	320 (75%)	320 (75%)	398 (100)	397 (100)	101 (60.8)	89 (53.6)
2 prior therapies	0	0	0	0	0	0	0	0	0	0	66 (19)	75 (22)	51 (35)	47 (33)			105 (25%)	105 (25%)	0	0	65 (39.2)	77 (46.4)

Nine studies [8, 9, 47–49, 51, 53, 54, 57] were considered at high risk of bias for PFS and OS (due to the lack of blinding of participants and personnel). The five RCTs [9, 47–49, 54] evaluating immunotherapies were open-label and therefore were rated as high-risk on the domain of performance bias.

The only study at low RoB for all the domains was LUME-LUNG 1. [10] The majority of studies were rated as high-risk on ‘other domains of bias’ due to being funded by industry (Supplementary online material B in S3 File).

There was no substantial imbalance in the distribution of the effect modifiers across studies in the networks. The dosages and administration modes of the anchored treatments across trials were consistent.

### Efficacy outcomes (overall analysis regardless of histology groups)

**Fig 2:**
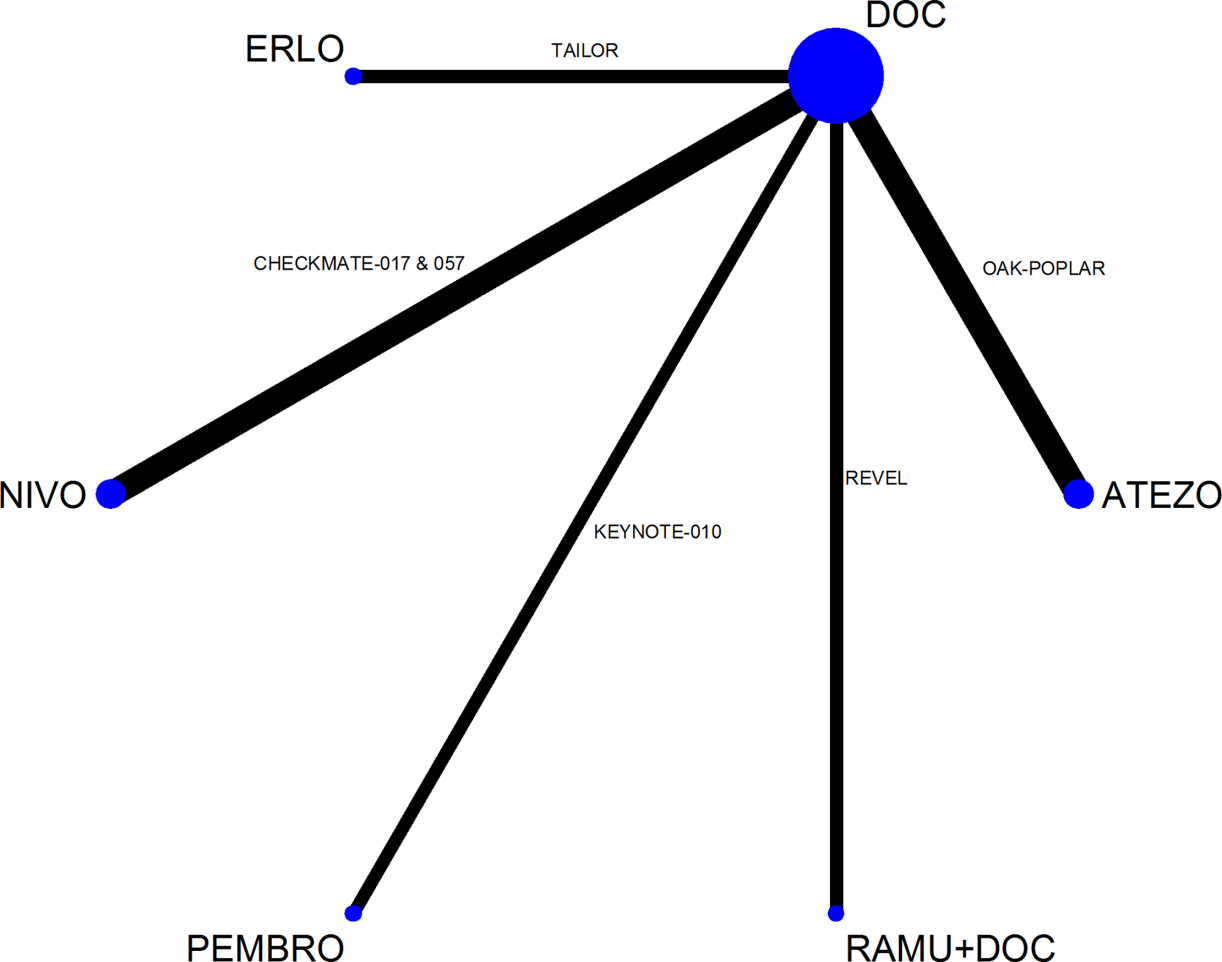
Network of studies comparing effectiveness (OS, PFS) and safety (grade 3–5 drug-related AE) outcomes in all-histology NSCLC.

The evidence formed a connected star-shaped network with only a single RCT for most of the comparisons (Fig 2). [8, 9, 50] Four included RCTs were not presented in the network plot because in these one of the evaluated interventions was restricted in its label indication to one specific histology subgroup (i.e. the intervention is not licenced for NSCLC irrespective of the patient’s tumour histology). [10, 51, 53, 57] These four RCTs were used in the analyses by histological subgroup the results of which are reported in the subsequent sections.

There was no evidence suggesting that the transitivity assumption was violated in any of the networks.

The inconsistency test was not conducted as planned due to the absence of closed loops in the network.

#### Overall survival

**Fig 3:**
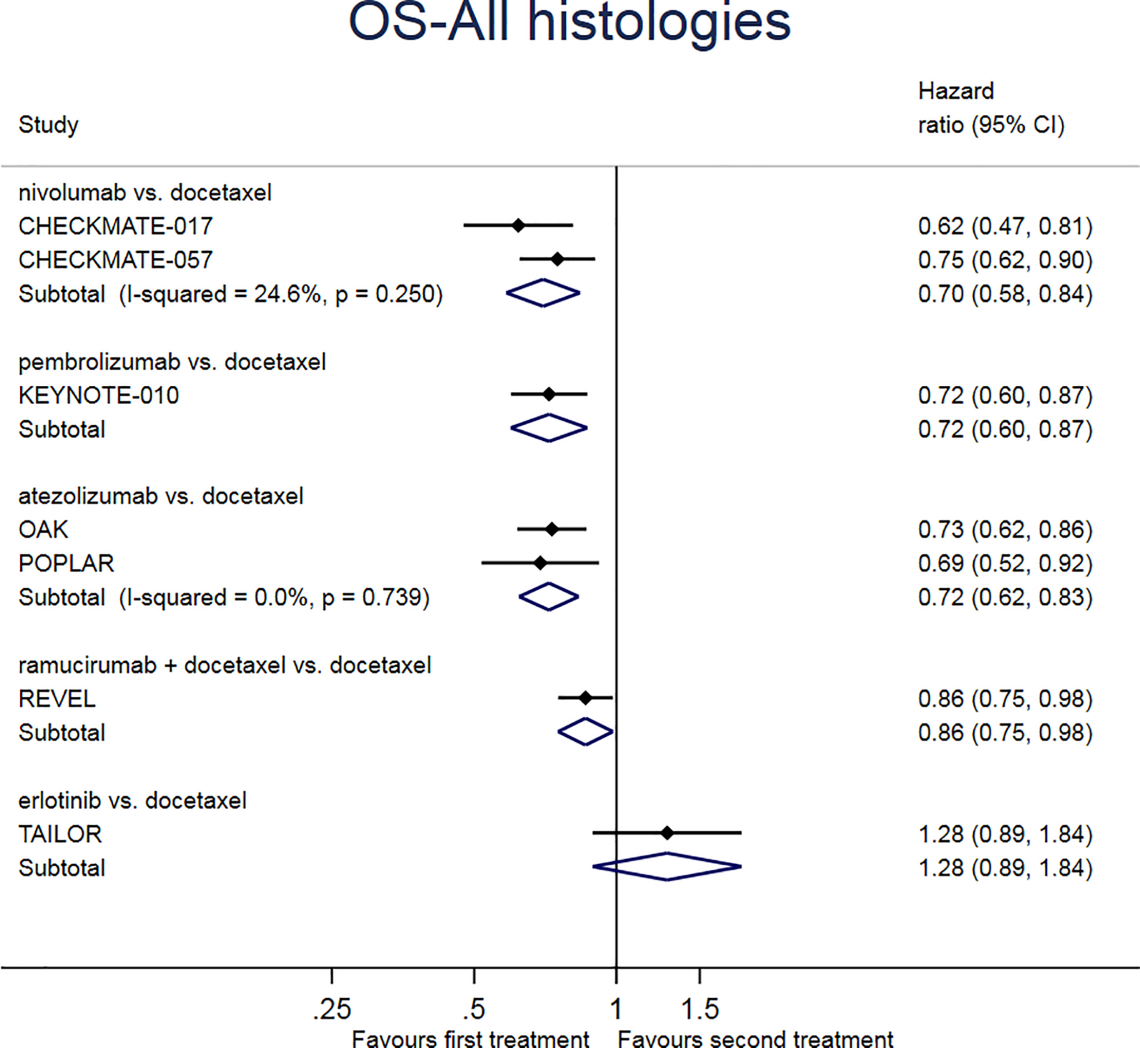
Pairwise meta-analyses, OS in all-histology NSCLC.

Four drugs (NIVO, ATEZO, PEMBRO, and RAMU+DOC) showed a significant improvement on OS compared to DOC in head-to-head comparisons (Fig 3). Indirect comparisons of drugs superior to DOC showed greater SUCRA values for the checkpoint inhibitors NIVO (0.82), ATEZO (0.77), PEMBRO (0.77) than for RAMU+DOC (0.42) (Table 2). There was no significant difference in OS across three highest ranking drugs (HR = 0.98, 95% CI 0.79, 1.21 for NIVO vs ATEZO; HR = 0.98, 95% CI 0.77, 1.25 for NIVO vs PEMBRO).

**Table 2:** Network meta-analyses: PFS, OS, grade 3–5 AE in all-histology NSCLC.

**OS comparisons *(Findings are expressed as HR (95% CI)*, *use of random-effects model*.**							
Drug	SUCRA	Nivo	Atezo	Pembro	Ramu+Doc	Doc	Erlo
Nivo	0.82		0.98 (0.79,1.21)	0.98 (0.77,1.25)	0.82 (0.67,1.00)	0.71 (0.61,0.82)	0.55 (0.37,0.82)
Atezo	0.77			1.00 (0.79,1.27)	0.84 (0.69,1.02)	0.72 (0.62,0.83)	0.56 (0.38,0.83)
Pembro	0.77				0.84 (0.67,1.05)	0.72 (0.60,0.87)	0.56 (0.37,0.85)
Ramu+Doc	0.42					0.86 (0.75,0.98)	0.67 (0.46,0.99)
Doc	0.18						0.78 (0.54,1.12)
Erlo	0.02						
**PFS comparisons (*Findings expressed as HR (95% CI)*, *use of random-effects model*.**							
Drug	SUCRA	Ramu+Doc	Nivo	Pembro	Atezo	Doc	Erlo
Ramu + Doc	0.84		0.98 (0.68,1.41)	0.86 (0.58,1.29)	0.80 (0.57,1.14)	0.76 (0.58,0.99)	0.55 (0.35,0.88)
Nivo	0.81			0.88 (0.60,1.29)	0.82 (0.59,1.13)	0.77 (0.61,0.99)	0.56 (0.36,0.88)
Pembro	0.57				0.93 (0.64,1.35)	0.88 (0.65,1.18)	0.64 (0.39,1.03)
Atezo	0.45					0.95 (0.76,1.18)	0.69 (0.44,1.06)
Doc	0.31						0.72 (0.50,1.06)
Erlo	0.02						
**Grade 3–5 AE comparisons *(Findings are expressed as RR (95% CI)*, *use of random-effects model*.**							
Drug	SUCRA	Nivo	Atezo	Pembro	Erlo	Doc	Ramu+Doc
Nivo	1		0.55 (0.38,0.79)	0.52 (0.34,0.81)	0.46 (0.29,0.72)	0.18 (0.14,0.25)	0.17 (0.12,0.23)
Atezo	0.68			0.95 (0.66,1.38)	0.83 (0.55,1.23)	0.34 (0.28,0.41)	0.31 (0.25,0.38)
Pembro	0.63				0.87 (0.54,1.39)	0.35 (0.26,0.48)	0.32 (0.23,0.44)
Erlo	0.49					0.41 (0.29,0.58)	0.37 (0.26,0.53)
Doc	0.2						0.91 (0.85,0.97)
Ramu+Doc	0						

Note: The table must be read as the drug on the column against the drug on the row. For example the PFS HR of ramucirumab+docetaxel against nivolumab is 0.98 (95%CI 0.68, 1.41).

#### Progression-free survival

In head-to-head comparisons, only RAMU+DOC showed a significant improvement in PFS compared to DOC (Supplementary online material C in S3 File). Only the RAMU+DOC vs ERLO and NIVO vs ERLO indirect comparisons reached statistical significance (Table 2). The SUCRA rankings suggested RAMU+DOC (0.84) as the best intervention followed by NIVO (0.81), PEMBRO (0.57), ATEZO (0.45), DOC (0.31) and ERLO (0.02) which ranked last.

#### Drug-related grade 3–5 adverse events

Direct comparisons (Supplementary online material D in S3 File) showed significantly reduced risk of drug-related grade 3–5 AE with NIVO, ATEZO, PEMBRO, and ERLO compared to DOC alone. The same drugs were associated with reduced risk of these AEs compared to RAMU+DOC in indirect comparisons (Table 2). The SUCRA values for the checkpoint inhibitors were higher (range: 0.63–1.00) than for ERLO (0.49). Of the three highest ranking drugs (NIVO, ATEZO, PEMBRO), the safety profile of NIVO was significantly better than that of ATEZO (RR = 0.55, 95% CI 0.38, 0.79) and PEMBRO (0.52, 95% CI 0.34, 0.81).

#### Discontinuation due to drug-related AE

No NMA could be conducted for this outcome, because unlike for the previous outcome (Supplementary online material E in S3 File) the RR estimates from direct comparisons were not stable across different points of study follow-up (Supplementary online material F in S3 File).

#### Overall results (cluster rank analysis)

**Fig 4:**
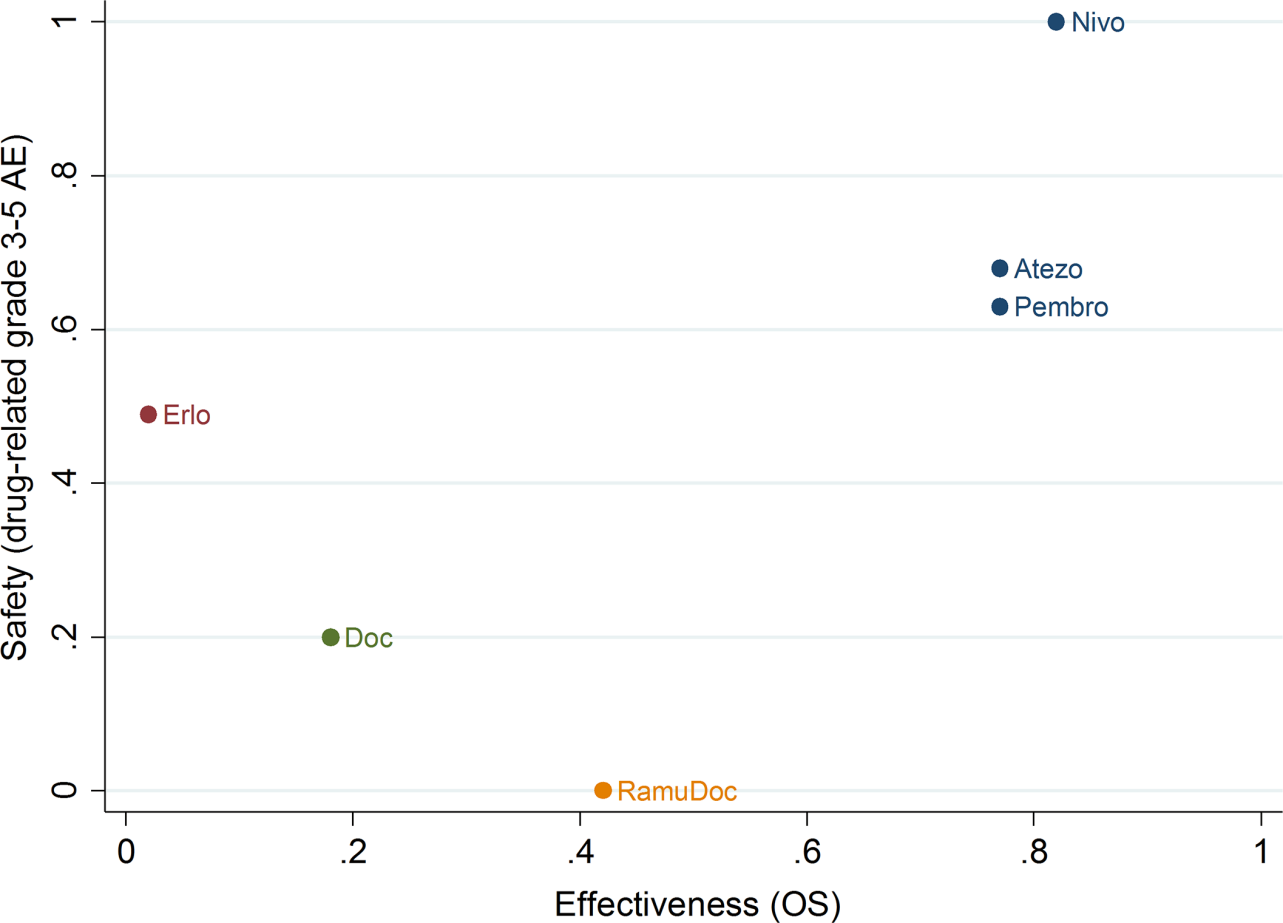
Clustered ranking plot on effectiveness (OS) and safety (grade 3–5 drug-related AE) both expressed as SUCRAS. Note: Y and X axes represent the cumulative ranking curve (SUCRA) to rank each intervention (i.e., probability between 0 to 1 of an intervention being superior in effectiveness or in safety compared to DOC); the plot guides a reader with respect to the trade-off between safety (measured drug-related grade 3–5 AE) and effectiveness (measures as OS) across the interventions: interventions in the right upper corner tend to be safer (higher SUCRA for AEs) and more effective (SUCRAs for OS) than those in the left lower corner of the plot (with lower SUCRAs on both factors). Thus, the Fig 3 supports a superior efficacy and safety for NIVO, ATEZO, and PEMBRO as opposed to DOC or ERLO. Also although NIVO compared to ATEZO and PEMBRO had similar effectiveness it appeared safer than the latter two.

Overall, NIVO, ATEZO and PEMBRO exhibited dominance in efficacy and safety over alternative therapies. According to the cluster rank analysis, NIVO was the drug with both the highest probability of being the most effective (overall survival) and the safest (drug-related grade 3–5 AEs) followed by ATEZO and PEMBRO (Fig 4).

### Efficacy outcomes by histology subgroups

The NMA for safety outcomes could not be performed due to sparse data.

#### Squamous histology

Head-to-head comparisons for OS and PFS are reported in Supplementary online materials G and H (both in S3 File), respectively. The studies formed connected, but sparse networks for OS and PFS, because not all studies reported these outcomes (Supplementary online material I in S3 File).

For OS, the SUCRA rankings suggested that NIVO (0.89) was the best intervention followed by ATEZO (0.72), PEMBRO (0.65), RAMU+DOC (0.42), AFA (0.46), DOC (0.20), with ERLO (0.16) ranking the last (Supplementary online material J in S3 File). Indirect comparison estimates between checkpoint drugs (PEMBRO, ATEZO, and NIVO) vs. each other or vs. RAM + DOC or AFA were not significantly different. For PFS, the SUCRA rankings suggested that NIVO (0.95) was the best intervention followed by RAMU+DOC (0.76), PEMBRO (0.61), DOC (0.41), and AFA (0.25), with ERLO (0.02) ranking the last (Supplementary online material K in S3 File).

#### Non-squamous histology

Direct comparison estimates for OS and PFS are reported in Supplementary online materials L and M, respectively with corresponding network plots in Supplementary online material N (in S3 File). Based on the SUCRA rankings for OS (Supplementary online material O in S3 File), checkpoint inhibitors (PEMBRO, ATEZO, and NIVO) were the best interventions (0.94, 0.75, and 0.67, respectively) followed by PEM (0.59), NINTE + DOC (0.46), RAMU+DOC (0.46), and DOC (0.15), with ERLO (0.0) ranking the last. Among the four drugs with the highest rankings on OS, no significant difference was observed.

For PFS, the network plot included one closed loop allowing a mixed treatment comparison between DOC, ERLO, and PEME (Supplementary online material N2 in S3 File). There was no evidence of inconsistency for the mixed treatment comparison (DOC, ERLO, PEME comparisons) within this loop (p = 0.07). The SUCRA rankings from the NMA suggested that RAMU+DOC (0.85) and NINTE+DOC (0.83) were the best interventions followed by PEMBRO (0.58) and NIVO (0.49), PEME (0.49), and DOC (0.16), with ERLO (0.10) ranking the last (Supplementary online material P in S3 File). Among the four drugs with the highest rankings on PFS, no significant difference was observed.

## Discussion

Overall, the evidence in this review indicated that the checkpoint inhibitors (NIVO, ATEZO, and PEMBRO) were superior in improving OS compared to non-immunotherapies irrespective of population histology (mixed, squamous or non-squamous) in people with advanced or metastatic NSCLC after failure to prior chemotherapy.

For PFS, the checkpoint inhibitors performed worse than RAM +DOC (in mixed and non-squamous groups) and NINTE + DOC (in non-squamous groups) but were superior to other interventions (AFA, ERLO, DOC +PEME + ERLO, PEME +DOC).

Indirect comparisons showed significantly reduced risks of drug-related grade 3–5 AEs with checkpoint inhibitors (NIVO, ATEZO, and PEMBRO) compared to RAMU+DOC. Taken together with OS results, this evidence suggested that the three immunotherapies were superior to other treatments (AFA, ERLO, PEME, DOC).

The occurrence of drug-related AE is a time-varying outcome so that intervention comparisons are best examined using similar periods of exposure/follow-up per patient. In included studies, safety outcomes were reported at different points of follow-up.

Results based on indirect comparisons suggested a significantly reduced risk of drug-related grade 3–5 AEs with NIVO vs. ATEZO or PEMBRO (through DOC as the common comparator). One explanation could be the non-uniform occurrence rate of these events in the DOC arms (range: 35.9% [52] to 58.1% [46]) even though the same licenced dose regimen was used and duration of DOC treatment was comparable across the studies. Baseline characteristics of included patients do not suggest a particular reason explaining these differences. The incidence of drug-related grade 3–5 AEs across immunotherapies arms also showed slight differences between the three immunotherapies (range: 7.6% for NIVO [48] to 14.8% for ATEZO [54]). Owing to the above-mentioned discrepancies and the limited number of trials for each comparison, the observed more favourable safety profile of NIVO should be viewed with caution.

Peng et al. [58] have previously reported similar results regarding the better safety profile of NIVO vs PEMBRO.

In this work focusing on wild-type NSCLC (ALK and EGFR expression predominantly or 100% negative), ERLO was included although the summary of product characteristics for this drug indicates that “no survival benefit or other clinically relevant effects of the treatment have been demonstrated in patients with EGFR negative tumours”. However, we included ERLO in our review, because we considered that the label indication does still theoretically include people with EGFR—expression.

In patients with squamous histology, NIVO and ATEZO were the only drugs significantly improving OS compared to DOC. Effectiveness of PEMBRO vs DOC was of similar as that of ATEZO vs DOC but the former was not statistically significant, one explanation for which could be lower statistical power in KEYNOTE-010 to show an OS benefit per histology. The higher ranking of NIVO compared to ATEZO and PEMBRO observed for OS could be explained by a lower rate of OS in the DOC arm in CHECKMATE-017 [48] compared to that in OAK [54] or in REVEL. [26] The low number of studies per comparison limited the interpretation of these findings. Although this subgroup analysis suggested the immunotherapies as the most effective for OS, there was little evidence showing one of the three drugs of this class being superior to another.

The meta-analyses in patients with non-squamous histology showed significantly improved OS with all the drugs except for ERLO compared to DOC. None of the indirect comparisons across PEMBRO, ATEZO, NIVO, PEME, NINTE+DOC and RAMU+DOC showed a significant improvement in OS. We were unable to meaningfully compare drugs on safety outcomes in the histology-specific subgroups of patients.

A recently published systematic review with NMA synthesised 102 RCTs to assess the efficacy and safety of 61 second-line treatments for patients with NSCLC regardless whether or not drugs (or drug combinations) were licensed or commercialised in this population. [59] Although the review authors provided a comprehensive evidence synthesis, their findings may have limited applicability to routine clinical practice. In contrast, the focus on licensed indications and dose regimens renders our review clinically more relevant.

Our work has several limitations. Although we used a systematic search approach we may have missed some unpublished relevant studies with null findings, so the potential for publication bias cannot be excluded. Because of the scarcity of evidence, we could not assess if RoB affected the NMA results due to either the lack of blinding or to industry sponsorship that potentially might influence some findings. Different definitions of safety outcomes and their reporting at different follow-ups may have affected the validity of drug comparisons. A further limitation is that in our NMA we used Cox regression model-based HR estimates that were stratified according to characteristics specified for randomisations, the use of which was not entirely consistent across the analysed studies.

In general, the differences in potential effect modifiers across studies were not substantial to violate the transitivity assumption.

The applicability of this review results may be limited owing to a changing landscape for the first-line treatment because immunotherapies are becoming standard treatments in this setting. This is particularly the case for PEMBRO which demonstrated improved survival outcomes compared to platinum-based chemotherapy in people with PD-L1 expression ≥50%. [60] Should PEMBRO become a standard care at first line, one can assume that people with PD-L1 expression ≥50% receiving PEMBRO at first-line and progressing will not receive subsequent lines of other immunotherapies. Therefore, our findings may not be applicable for people with PD-L1 expression ≥50% (around 30% of NSCLC [60]).

## Conclusions

In this review, we advanced the existing knowledge by comparing drugs approved in people with non-specific late-stage NSCLC. Our results indicate that the use of immunotherapies in people diagnosed with non-specific late stage NSCLC should be promoted. Amongst our included studies, more than 3,500 patients received licensed dosing of DOC, which proved relatively unsuccessful on both survival and safety. The use of DOC may now be judged irrelevant as a comparator intervention for approval of new drugs for second line treatment of NSCLC.

## Supporting information

S1 File Study protocol registered in PROSPERO. (PDF)

S2 File Prisma checklist. (DOC)

S3 File Supplemental appendix: Content. Supplementary online material A: Medline search strategy Supplementary online material B: Risk of bias assessment Supplementary online material C: Pairwise meta-analyses, PFS in all-histology NSCLC Supplementary online material D: Pairwise meta-analyses, grade 3–5 AE related to drugs in all-histology NSCLC Supplementary online material E: Pairwise meta-analyses, grade 3–5 AE related to drugs in all-histology NSCLC according to follow-up duration Supplementary online material F: Pairwise meta-analyses, discontinuation due to drug-related AE in all-histology NSCLC according to follow-up duration Supplementary online material G: Pairwise meta-analyses, OS in squamous NSCLC Supplementary online material H: Pairwise meta-analyses, PFS in squamous histology Supplementary online material I: Network of studies, OS (a) and PFS (b) in squamous histologies Supplementary online material J: Network meta-analysis: OS in Squamous NSCLC Supplementary online material K: Network meta-analysis: PFS in squamous NSCLC Supplementary online material L: Pairwise meta-analyses, OS in non-squamous NSCLC Supplementary online material M: Pairwise meta-analyses, PFS in non-squamous histology Supplementary online material N: Network of studies, OS (1) and PFS (2) in non-squamous histology Supplementary online material O: Network meta-analysis: OS in non-squamous NSCLC Supplementary online material P: Network meta-analysis: PFS in non-squamous NSCLC.
(DOCX)

S4 File Data underlying our study. These correspond to data extracted from the primary research papers which were subsequently used in meta-analyses.
(XLSX)
