# Supplementary material for: Comparative efficacy and safety of licensed treatments for previously treated non-small cell lung cancer: A systematic review and network meta-analysis
Source: PLoS One. 2018 Jul 25;13(7):e0199575. doi: 10.1371/journal.pone.0199575 (PMC6059384; doi:10.1371/journal.pone.0199575)
Supplement: S1 File — (PDF) [file pone.0199575.s001.pdf]

NHS
National Institute for Health Research

The comparative effectiveness and safety of currently licensed treatments for patients with advanced or metastatic non-small cell lung cancer after the failure of prior platinum-based regimens: a systematic review and network meta-analysis

Xavier Armoiry, Alexander Tsertsvadze, G.J. Melendez-Torres, Pamela Royle, Aileen Clarke, Martin Connock

Citation

Xavier Armoiry, Alexander Tsertsvadze, G.J. Melendez-Torres, Pamela Royle, Aileen Clarke, Martin Connock. The comparative effectiveness and safety of currently licensed treatments for patients with advanced or metastatic non-small cell lung cancer after the failure of prior platinum-based regimens: a systematic review and network meta-analysis. PROSPERO 2017 CRD42017065928 Available from:
http://www.crd.york.ac.uk/PROSPERO/display_record.php?ID=CRD42017065928

Review question

To compare the effectiveness and safety of currently licensed drugs in patients with NSCLC who have failed to respond to the first-line treatment of platinum-based therapy.

Searches

Electronic databases (MEDLINE; EMBASE; Web of Science) will be searched for relevant literature from 2000 up to present.
The electronic searches will be limited to English language only.

Types of study to be included

All randomised controlled trials (RCTs), with no minimum follow-up duration.

Condition or domain being studied

Second-line treatment for non-small cell lung cancer.

Participants/population

Adults with advanced or metastatic (IIIB and/or IV) NSCLC will be included:
1) Non-squamous (adenocarcinoma, large cell), or squamous histology;
2) Anaplastic lymphoma kinase (ALK) expression either predominantly negative or 100% negative;
3) Epidermal growth factor receptor (EGFR) expression either predominantly negative or 100% negative;
4) Patients who failed to respond to prior first-line chemotherapy (i.e., those receiving second-line treatment or beyond).

Intervention(s), exposure(s)

All drugs with a label indication (EMA marketing authorisation or CHMP positive opinion) that covers, as of May, 2017, the population described above.
The drugs meeting these criteria are listed here:
- Docetaxel (DOC);
- Pemetrexed (PEM);
- Ramucirumab in combination with docetaxel (RAM + DOC);
- Erlotinib (ERL);
- Nintedanib in combination with docetaxel (NIN + DOC);
- Afatinib (AFA);
- Nivolumab (NIVO);
- Pembrolizumab (PEMBRO);
- Atezolizumab (ATEZO).

Comparator(s)/control

- Any other active treatment alone or in combination with a drug of interest (e.g., standard chemotherapy).
- Best supportive care alone (non-active treatment) or in combination with a drug of interest.

Primary outcome(s)

Overall survival (OS).
Progression-free survival (PFS).

Secondary outcome(s)

Proportion of patients reporting at least one grade 3 & 4 adverse event (AE).
Proportion of patients discontinuing study medication due to AE.

Data extraction (selection and coding)

Using a pre-piloted screening form, three reviewers will independently screen all titles/abstracts and then full texts of publications potentially relevant for inclusion in the review. Any disagreements will be discussed and resolved through a consensus or with help from a third reviewer. The study flow and reasons for exclusion at the full text screening level will be documented and presented in the PRISMA study flow diagram (Moher 2015).
Two reviewers will independently extract relevant data using an a priori defined pre-piloted extraction sheet. The extracted data will be cross-checked and any disagreements will be resolved by discussion or by recourse to a third party reviewer.

Risk of bias (quality) assessment

Two independent reviewers will assess the risk of bias (RoB) of direct comparison evidence in included studies (per outcome: OS and PFS) using the Cochrane RoB tool for RCTs (Higgins 2011).

Strategy for data synthesis

Study, intervention, population, and outcome characteristics will be summarised in text, evidence, and summary tables. Results of individual studies will also be provided.
Where possible, we will undertake a network meta-analysis of interventions on both effectiveness and safety outcomes using a frequentist approach
Where possible, and using IPD reconstructed from Kaplan-Meier plots, we will estimate an IPD-based network meta-analysis for relevant outcomes (survival outcomes).

Analysis of subgroups or subsets

Study, intervention, population, and outcome characteristics will be summarised in text, evidence, and summary tables. Results of individual studies will also be provided.
Where possible, the analyses will be stratified by histologic subtypes (squamous and non-squamous) and tumour stage.

Contact details for further information

Dr Armoiry
X.Armoiry@warwick.ac.uk

Organisational affiliation of the review

The University of Warwick

Review team members and their organisational affiliations

Dr Xavier Armoiry.
Dr Alexander Tsertsvadze.
Dr G.J. Melendez-Torres.
Dr Pamela Royle.
Professor Aileen Clarke.
Dr Martin Connock.

Anticipated or actual start date
12 May 2017

Anticipated completion date
01 September 2017

Funding sources/sponsors
None

Conflicts of interest
None known

Language
English

Country
England

Stage of review
Review_Ongoing

Subject index terms status
Subject indexing assigned by CRD

Subject index terms
Carcinoma, Non-Small-Cell Lung; Humans; Licensure; Lung Neoplasms; Network Meta-Analysis; Safety

Date of registration in PROSPERO
15 May 2017

Date of publication of this version
05 July 2017

Revision note for this version
We have clarified the inclusion criteria to reflect the fact that we are interested in treatments for advanced NSCLC after prior platinum-based therapy, which corresponds to second-line treatments and beyond, and not just to second-line treatments alone.

Details of any existing review of the same topic by the same authors

Stage of review at time of this submission

Stage	Started	Completed
Preliminary searches	Yes	No
Piloting of the study selection process	Yes	No
Formal screening of search results against eligibility criteria	No	No
Data extraction	No	No
Risk of bias (quality) assessment	No	No
Data analysis	No	No

National Institute for Health Research

Revision note

We have clarified the inclusion criteria to reflect the fact that we are interested in treatments for advanced NSCLC after prior platinum-based therapy, which corresponds to second-line treatments and beyond, and not just to second-line treatments alone.

Versions

15 May 2017
05 July 2017

PROSPERO
This information has been provided by the named contact for this review. CRD has accepted this information in good faith and registered the review in PROSPERO. CRD bears no responsibility or liability for the content of this registration record, any associated files or external websites.
