# Supplementary material for: Comparative efficacy and safety of licensed treatments for previously treated non-small cell lung cancer: A systematic review and network meta-analysis
Source: PLoS One. 2018 Jul 25;13(7):e0199575. doi: 10.1371/journal.pone.0199575 (PMC6059384; doi:10.1371/journal.pone.0199575)
Supplement: S3 File — Supplementary online material A: Medline search strategy Supplementary online material B: Risk of bias assessment Supplementary online material C: Pairwise meta-analyses, PFS in all-histology NSCLC Supplementary online material D: Pairwise meta-analyses, grade 3–5 AE related to drugs in all-histology NSCLC Supplementary online material E: Pairwise meta-analyses, grade 3–5 AE related to drugs in all-histology NSCLC according to follow-up duration Supplementary online material F: Pairwise meta-analyses, discontinuation due to drug-related AE in all-histology NSCLC according to follow-up duration Supplementary online material G: Pairwise meta-analyses, OS in squamous NSCLC Supplementary online material H: Pairwise meta-analyses, PFS in squamous histology Supplementary online material I: Network of studies, OS (a) and PFS (b) in squamous histologies Supplementary online material J: Network meta-analysis: OS in Squamous NSCLC Supplementary online material K: Network meta-analysis: PFS in squamous NSCLC Supplementary online material L: Pairwise meta-analyses, OS in non-squamous NSCLC Supplementary online material M: Pairwise meta-analyses, PFS in non-squamous histology Supplementary online material N: Network of studies, OS (1) and PFS (2) in non-squamous histology Supplementary online material O: Network meta-analysis: OS in non-squamous NSCLC Supplementary online material P: Network meta-analysis: PFS in non-squamous NSCLC. (DOCX) [file pone.0199575.s003.docx]

***Online appendices – Supplementary material***

**Supplementary online material A: Medline search strategy**

1. (docetaxel or pemetrexed or ramucirumab or erlotinib or nintedanib or afatinib or nivolumab or pembrolizumab or atezolizumab).tw.

2. (non-small cell lung cancer or nsclc).tw.

3. (squamous cell adj4 lung adj4 (cancer or carcinoma)).tw.

4. *Carcinoma, Non-Small-Cell Lung/dt [Drug Therapy]

5. *Carcinoma, Squamous Cell/dt [Drug Therapy]

6. 2 or 3 or 4 or 5

7. 1 and 6

8. (random* or double blind or phase 3 or phase III).tw.

9. randomized controlled trial.pt.

10. 8 or 9

11. 7 and 10

12. limit 11 to english language

13. limit 12 to yr="2000 -Current"

14. (letter or comment or editorial).pt.

15. 13 not 14

**Supplementary online material B: Risk of bias assessment**

1. **Methods**

The following domains were assessed: selection bias (random sequence generation, allocation concealment), performance bias (blinding participants and personnel), detection bias (blinding of outcome assessment), attrition bias (incomplete outcome data), reporting bias (selective outcome reporting), and other bias (between-group baseline distribution of important prognostic factors, study sponsor). We used the domain “other risk of bias” to indicate RCTs where a drug manufacturer was a sponsor as at high risk of bias.

For each outcome, a summary rating of high RoB was assigned if at least one of the domains of selection, attrition, and other bias was rated as high RoB. A summary rating of low RoB was assigned if all domains of bias were rated as low RoB. An outcome was assigned as an unclear RoB rating if the given information was insufficient to judge the corresponding RoB. Quality assessment was performed by two independent reviewers (X.A. & A.T.) and these assessments were cross-checked. Any disagreements between reviewers were resolved by a third reviewer (M.C.) through a discussion.

1. **Risk of bias assessment for each study (PFS as an outcome)**

1. **Risk of bias assessment across domains (PFS as an outcome)**
2. **Risk of bias assessment for each study (OS as an outcome)**

1. Risk of bias assessment across domains (OS as an outcome)

**Supplementary online material C: Pairwise meta-analyses, PFS in all-histology NSCLC**

**Supplementary online material D: Pairwise meta-analyses, grade 3-5 AE related to drugs in all-histology NSCLC**

**Supplementary online material E: Pairwise meta-analyses, grade 3-5 AE related to drugs in all-histology NSCLC according to follow-up duration**

**Supplementary online material F: Pairwise meta-analyses, discontinuation due to drug-related AE in all-histology NSCLC according to follow-up duration**

**Supplementary online material G: Pairwise meta-analyses, OS in squamous NSCLC**

**Supplementary online material H: Pairwise meta-analyses, PFS in squamous histology**

**Supplementary online material I: Network of studies, OS (a) and PFS (b) in squamous histologies**

**(1)**

**(2)**

**Supplementary online material J: Network meta-analysis: OS in Squamous NSCLC**

| Drug | SUCRA | Nivo | Atezo | Pembro | Ramu-Doc | Afa | Doc | Erlo |
| --- | --- | --- | --- | --- | --- | --- | --- | --- |
| Nivo | 0.89 |  | 0.87 (0.61,1.26) | 0.84 (0.52,1.34) | 0.70 (0.49,1.01) | 0.68 (0.35,1.34) | 0.62 (0.47,0.81) | 0.56 (0.29,1.08) |
| Atezo | 0.72 |  |  | 0.96 (0.60,1.52) | 0.81 (0.57,1.15) | 0.78 (0.40,1.53) | 0.71 (0.55,0.91) | 0.64 (0.33,1.23) |
| Pembro | 0.65 |  |  |  | 0.84 (0.53,1.33) | 0.81 (0.39,1.70) | 0.74 (0.50,1.09) | 0.67 (0.32,1.37) |
| Ramu-Doc | 0.42 |  |  |  |  | 0.97 (0.49,1.89) | 0.88 (0.69,1.13) | 0.79 (0.41,1.52) |
| Afa | 0.46 |  |  |  |  |  | 0.91 (0.49,1.70) | 0.82 (0.70,0.96) |
| Doc | 0.2 |  |  |  |  |  |  | 0.90 (0.49,1.65) |
| Erlo | 0.16 |  |  |  |  |  |  |  |

***Findings expressed as HR (95% CI), use of random-effects model.***

**Supplementary online material K: Network meta-analysis: PFS in squamous NSCLC**

| Drug | SUCRA | Nivo | Ramu- Doc | Pembro | Doc | Afa | Erlo |
| --- | --- | --- | --- | --- | --- | --- | --- |
| Nivo | 0.95 |  | 0.83 (0.58,1.18) | 0.73 (0.48,1.12) | 0.63 (0.48,0.83) | 0.44 (0.23,0.86) | 0.36 (0.19,0.69) |
| Ramu-Doc | 0.76 |  |  | 0.88 (0.59,1.32) | 0.76 (0.61,0.95) | 0.53 (0.28,1.01) | 0.43 (0.23,0.81) |
| Pembro | 0.61 |  |  |  | 0.86 (0.62,1.20) | 0.60 (0.30,1.20) | 0.49 (0.25,0.96) |
| Doc | 0.41 |  |  |  |  | 0.70 (0.38,1.28) | 0.57 (0.32,1.03) |
| Afa | 0.25 |  |  |  |  |  | 0.82 (0.70,0.96) |
| Erlo | 0.02 |  |  |  |  |  |  |

***Findings expressed as HR (95% CI), use of fixed-effects model.***

**Supplementary online material L: Pairwise meta-analyses, OS in non-squamous NSCLC**

**Supplementary online material M: Pairwise meta-analyses, PFS in non-squamous histology**

** the HR estimate of the HORG study corresponds to Time To Progression which was used as a proxy for the Progression Free Survival HR*

**Supplementary online material N: Network of studies, OS (a) and PFS (b) in non-squamous histology**

**(1)**

**(2)**

**Supplementary online material O: Network meta-analysis: OS in non-squamous NSCLC**

| Drug | SUCRA | Pembro | Atezo | Nivo | Peme | Ninte-Doc | Ramu-Doc | Doc | Erlo |
| --- | --- | --- | --- | --- | --- | --- | --- | --- | --- |
| Pembro | 0.94 |  | 0.88 (0.66,1.17) | 0.84 (0.63,1.13) | 0.81 (0.58,1.13) | 0.76 (0.57,1.01) | 0.76 (0.57,1.00) | 0.63 (0.50,0.79) | 0.42 (0.28,0.64) |
| Atezo | 0.75 |  |  | 0.96 (0.75,1.24) | 0.92 (0.68,1.25) | 0.87 (0.68,1.11) | 0.87 (0.69,1.09) | 0.72 (0.61,0.86) | 0.48 (0.33,0.71) |
| Nivo | 0.67 |  |  |  | 0.96 (0.71,1.31) | 0.90 (0.70,1.16) | 0.90 (0.71,1.15) | 0.75 (0.62,0.90) | 0.50 (0.34,0.74) |
| Peme | 0.59 |  |  |  |  | 0.94 (0.70,1.27) | 0.94 (0.70,1.26) | 0.78 (0.61,1.00) | 0.52 (0.34,0.80) |
| Ninte-Doc | 0.46 |  |  |  |  |  | 1.00 (0.79,1.26) | 0.83 (0.70,0.99) | 0.56 (0.38,0.82) |
| Ramu-Doc | 0.46 |  |  |  |  |  |  | 0.83 (0.71,0.97) | 0.56 (0.38,0.81) |
| Doc | 0.15 |  |  |  |  |  |  |  | 0.67 (0.48,0.94) |
| Erlo | 0 |  |  |  |  |  |  |  |  |

***Findings expressed as HR (95% CI), use of random-effects model.***

**Supplementary online material P: Network meta-analysis: PFS in non-squamous NSCLC**

| Drug | SUCRA | Ramu-Doc | Ninte-Doc | Pembro | Nivo | Peme | Doc | Erlo |
| --- | --- | --- | --- | --- | --- | --- | --- | --- |
| Ramu-Doc | 0.85 |  | 1.00 (0.77,1.30) | 0.90 (0.71,1.13) | 0.87 (0.69,1.08) | 0.86 (0.67,1.08) | 0.77 (0.67,0.88) | 0.73 (0.55,0.96) |
| Ninte-Doc | 0.83 |  |  | 0.90 (0.67,1.19) | 0.87 (0.65,1.15) | 0.86 (0.64,1.15) | 0.77 (0.62,0.96) | 0.73 (0.53,1.00) |
| Pembro | 0.58 |  |  |  | 0.97 (0.75,1.25) | 0.96 (0.73,1.25) | 0.86 (0.71,1.04) | 0.81 (0.60,1.10) |
| Nivo | 0.49 |  |  |  |  | 0.99 (0.76,1.29) | 0.89 (0.74,1.06) | 0.84 (0.62,1.13) |
| Peme | 0.49 |  |  |  |  |  | 0.90 (0.74,1.09) | 0.85 (0.68,1.06) |
| Doc | 0.16 |  |  |  |  |  |  | 0.94 (0.74,1.20) |
| Erlo | 0.1 |  |  |  |  |  |  |  |

***Findings expressed as HR (95% CI), use of fixed-effects model.***
